# Persistent hypertension among postpartum women with comorbid HIV and preeclampsia in Zambia

**DOI:** 10.1371/journal.pone.0309915

**Published:** 2024-09-04

**Authors:** Moses Mukosha, Abigail Hatcher, Mwansa Ketty Lubeya, Innocent Maposa, Benjamin H. Chi, Wilbroad Mutale

**Affiliations:** 1 Faculty of Health Sciences, University of the Witwatersrand, Johannesburg, South Africa; 2 Department of Obstetrics and Gynaecology, University of Zambia, Lusaka, Zambia; 3 Department of Global Health, Division of Epidemiology & Biostatistics, Stellenbosch University, Cape Town, South Africa; 4 Department of Obstetrics and Gynecology, University of North Carolina at Chapel Hill, Chapel Hill, NC, United States of America; 5 Department of Health Policy and Management, University of Zambia, Lusaka, Zambia; University of Medical Sciences Ondo City, NIGERIA

## Abstract

**Background:**

Persistent hypertension is common after preeclampsia and is causally tied to later cardiovascular risks. This study examined whether being HIV-infected and on antiretroviral therapy (ART) is associated with persistent postpartum hypertension among women diagnosed with preeclampsia.

**Methods:**

We conducted a six-month prospective cohort study at Kanyama and Women and Newborn hospitals from January 01, 2022, to June 30, 2023, among 190 women diagnosed with preeclampsia (59 HIV-positive, 131 HIV-negative). Sociodemographic and clinical characteristics were collected at delivery, six weeks, three months and six months after giving birth. Persistent hypertension was diagnosed if a participant presented with elevated blood pressure ≥140mmHg and/or diastolic blood pressure ≥90mmHg and/or taking medication for hypertension at the study visit. We used a generalized estimating equation to describe the relationship between treated HIV and persistent hypertension six months following delivery.

**Results:**

We retained 136 participants (71.6%) to six months postpartum, at a median age of 30 years. Overall, persistent hypertension at six weeks, three months, and six months postpartum was common (37.4%, 17.1% and 16.9%, respectively). Six-week postpartum prevalence was higher in the HIV group than HIV-negative group (54.6% vs 28.8%, p<0.001), with no measurable difference at three months (24.3% vs 13.2%, p = 0.145) or six months (18.2% vs 16.3%, p = 0.787). Multivariable analysis demonstrates higher odds (adjusted odds ratio [aOR] = 1.68, 95% CI: 1.09–2.60) of persistent hypertension among the HIV+treatment group than HIV-negative counterparts after accounting for age, body mass index and time since delivery.

**Conclusion:**

We demonstrate an elevated risk of persistent hypertension among postpartum women with comorbid preeclampsia and treated HIV. Peripartum patients in HIV-endemic settings may benefit from timely detection of hypertension and treatment interventions to improve health outcomes.

## Introduction

Hypertension is a major cause of premature deaths globally, affecting nearly one in five women [1]. Hypertension is characterized by elevated systolic blood pressure ≥140mmHg and/or diastolic blood pressure ≥90mmHg or taking medication for hypertension [2]. The burden is shared disproportionately in low and middle-income countries (LMICs), where hypertension is estimated between 15% and 54% [3]. Most women in LMICs continue to die from preventable hypertension-related complications, such as stroke and ischaemic heart disease [4, 5]. In sub-Saharan Africa 42% of deaths are attributable to high blood pressure, of which 20% are female deaths [6].

Emerging evidence suggests that women with preeclampsia are at an increased risk of persistent hypertension and other cardiovascular diseases after giving birth and beyond [7–9]. There are many risk factors for persistent hypertension, including increased body mass index, the severity of preeclampsia, parity, and advanced maternal age [10, 11]. Other factors may be HIV and ART, although previous reports have not been conclusive [12, 13]. Small studies of women living with HIV reported a high prevalence of persistent hypertension (85.7% and 83.3%) [8, 14]. One plausible mechanism through which HIV and persistent hypertension could be associated is through the shared inflammation cascade in women recovering from preeclampsia [15–18]. Both HIV and preeclampsia are characterized by increased expression of pro-inflammatory cytokines which are responsible for endothelial dysfunction leading to hypertension [19, 20]. Additionally, there is evidence that suggests that ART regimens (i.e. protease inhibitors) may be associated with hypertension in pregnancy as well [21].

Furthermore, studies done during pregnancy suggest that HIV and ART are associated with worse health outcomes for women with preeclampsia, such as premature births, stillbirths and small for gestational age [22–25]. However, the association between persistent hypertension and HIV after preeclampsia remains unclear. Therefore, to address this gap, we examined whether being HIV-infected and on ART is associated with persistent postpartum hypertension six months after giving birth in women whose pregnancy was complicated by preeclampsia.

## Materials and methods

### Study design, setting and population

A prospective cohort study was conducted between Jan 01, 2022, and June 30, 2023, at the Women and Newborn Hospital and the Kanyama General Hospital in Lusaka Urban, Zambia. The two hospitals have obstetricians/gynaecologists and nurse midwives and receive referrals of women with preeclampsia from surrounding clinics. The health services are offered free of user fees. From the two hospitals on average about 24000 pregnant women are seen per year with over 12000 births.

Women diagnosed with preeclampsia were invited to participate after giving birth. Preeclampsia, in this setting, is diagnosed following the American College of Obstetricians and Gynecologists guidelines as a new-onset of hypertension (SBP ≥140 mm Hg and/or DBP ≥90 mm Hg) after 20 weeks of gestation and the presence of proteinuria or other maternal organ dysfunction [26]. The local hospital obstetrician diagnosed preeclampsia during antenatal clinic visits or at admission for referral cases, and the study staff verified the diagnostic criteria via independent chart reviews.

Antenatal care is offered routinely using the WHO 2016 guidelines and local guidelines. In tertiary and general hospitals, medical doctors are in charge of reviewing clients, while in primary healthcare facilities, midwives offer antenatal care, who then refer women with abnormal blood pressure readings and urinalysis for further evaluation. Women are offered a recommended eight antenatal visits. On each one of these visits, routine urinalysis and blood pressure measurements are taken.

We excluded women with a documented medical history of co-morbidities: diabetes mellitus, essential hypertension or taking antihypertensive medication, chronic renal failure, thyroid disorders and lupus erythematosus. This study has been reported following the Guidelines for Strengthening The Reporting of Observational Studies in Epidemiology (S1 Checklist) [27].

### Study procedures

From our prior work at the study site [28], we anticipated low numbers of women with HIV and preeclampsia. Therefore, we consecutively enrolled women living with HIV and enrolled HIV-negative participants using a systematic sampling technique based on average daily hospital deliveries and preeclampsia prevalence we enrolled every third eligible woman as indicated by entrance into the facility log book. At enrollment, we recorded contact information and made appointments for the remaining follow-up visits. Upon discharge from the hospital, participants were invited to return at six weeks, three months and six months postpartum. If there was elevated blood pressure at any visit, a follow-up visit was made 1–2 weeks later to confirm medical records for diagnosis of chronic hypertension. Women diagnosed with chronic hypertension were referred to the outpatient medical clinic for management.

### Study outcome

The primary study outcome was the proportion of women with persistent hypertension (Systolic Blood Pressure [SBP] ≥140 mmHg and/or Diastolic Blood Pressure [DBP] ≥90 mmHg at rest or receiving hypertension treatment) in the six months from delivery [5]. Blood pressure was measured on the day following delivery, six weeks, three months and six months. Research assistants measured blood pressure at least two times 5 minutes apart. For any measurements that differed by at least 10%, then a third measurement was done to confirm the final reading. Blood pressure measurements were taken after five minutes of rest in a seated upright position. We used a validated device for pregnancy and hypertensive pregnancy disorders so that accurate readings are obtained and utilized for clinical decisions [29]. Cuff sizes were made available during measurements based on the patient’s arm’s size, measured at the upper arm’s midpoint.

### Study exposures

The primary exposure of interest was HIV-serostatus. Since all pregnant women with HIV in this setting are offered ART (and since treatment uptake was high), the exposure was combined HIV+ART. Based on the Zambian clinical guidelines on HIV testing during pregnancy, all the women presenting to the antenatal clinic are continually tested as a routine standard of care [30]. Therefore, this study used maternal clinical records at enrollment to check for the HIV test results, and for those with missing results, the midwives conducted HIV tests after counselling using standard routine clinical practice guidelines [31, 32]. In addition, women who test positive are initiated on ART regardless of their immune status or clinical stage [32]. Therefore, all the participants in this study who were living with HIV were on ART. Currently, in Zambia, pregnant women are given a fixed combination of Tenofovir, Lamivudine and Dolutegravir as the preferred first-line treatment for HIV infection [33].

A structured questionnaire was offered by a trained nurse to assess socio-demographic characteristics of marital status (unmarried/married), maternal age (age in years from last birthday) and primary school education vs. higher levels of education (none, primary, secondary, tertiary). Additionally, we assessed food insecurity score using the household hunger scale [34] and socio-economic status using a demographic and health survey questionnaire with various economic characteristics (such as the source of drinking water, type of toilet, floor, roof, wall, cooking fuel and material possessions like television, radio etc) [35]. From the demographic and health survey questionnaire, scores were computed from all the ’YES’ answers and then categorised into quintiles of wealth index (poorest, poorer, middle, richer, richest). Body Mass Index (BMI) was measured from the participant’s weight and height, while gestational age was assessed based on the last menstrual period [31]. We also measured the severity of preeclampsia; mild (hypertension [140/90 to 149/99 mmHg]) moderate (150/100 to 159/109 mmHg) and severe (160/110 mmHg or higher) [36]. Other variables measured were the onset of preeclampsia, i.e., early-onset (less than 34 weeks gestation) and late-onset (more than 34 weeks gestation) [37], adverse pregnancy outcomes i.e., preterm birth (<37 completed weeks gestation) or stillbirth, Contraceptive use (Intrauterine Contraception, hormonal methods, barrier methods and dual methods) and probable anxiety/depression using validated cut points for the Hospital Depression and Anxiety Scale [38]. The scoring of anxiety and depression levels was done from 0 to 21 and categorised as follows; 0–9 no anxiety/depression and 10 or more, anxiety/depression.

### Sample size

We assumed the risk of persistent hypertension to be 35.0% in women living with HIV on ART versus 12% in HIV-negative women [14] and estimated that we would require at least 123 women (41 HIV on ART versus 82 HIV negative) to detect a 23% difference in risk of persistent hypertension with over 80% power and an alpha level of 5%. To meet this threshold while accounting for presumed loss to follow-up of over 20%, we enrolled 190 participants.

### Data management and analysis

We used both descriptive and analytical methods for statistical analysis. We described the change in hypertension by HIV serostatus using a bar graph to illustrate trends for the study population over time. We used the Pearson Chi-square test to assess the association between HIV and hypertension.

Using a Generalized Estimating Equation for panel data, we examined the effects of HIV and ART on the change in hypertension status (from hypertension to healthy and back to hypertension) over the six-month follow-up. We fitted both unadjusted and adjusted models with unstructured correlation. In the adjusted model, HIV, age and body mass index were set *a priori* based on the clinical importance. Other covariates were selected based on clinical relevance and statistical significance p<0.2 from the unadjusted model. In the final model interaction between time since delivery and HIV/ART was assessed at a significance level of alpha less than 0.05. The analysis used the intention-to-treat approach.

We used REDCap (Research Electronic Data Capture) electronic data capture tools hosted at the University of the Witwatersrand Johannesburg, South Africa, for data management and Stata (version 17/BE; StataCorp, College Station, Texas, USA) for data analysis. Statistical significance for all tests was set at p<0.05.

### Ethical consideration

We obtained ethical approval from the University of Zambia Biomedical Research Ethics Committee (ref: 1785–2021) and the Human Research Ethics Committee (Medical [ref: R14/49]) at the University of Witwatersrand School of Health Sciences South Africa. All participants provided fully written informed consent before enrollment. All information was explained to potential participants using non-technical language, in the participant’s preferred language. The confidentiality of all study records was safeguarded to the extent legally possible. All laboratory specimens, reports, study data and administrative forms were de-identified and the link log was stored on a password-protected online / computer database. Follow-up information about participants was collected separately and stored in lockable cupboards by the research assistants. Participants were allowed to discontinue participation and decide that their medical data could not be used in the analysis without repercussions on their medical care.

### Inclusivity in global research

Additional information regarding the ethical, cultural, and scientific considerations specific to inclusivity in global research is included in the Supporting Information (S2 Checklist)

## Results

**Fig 1:**
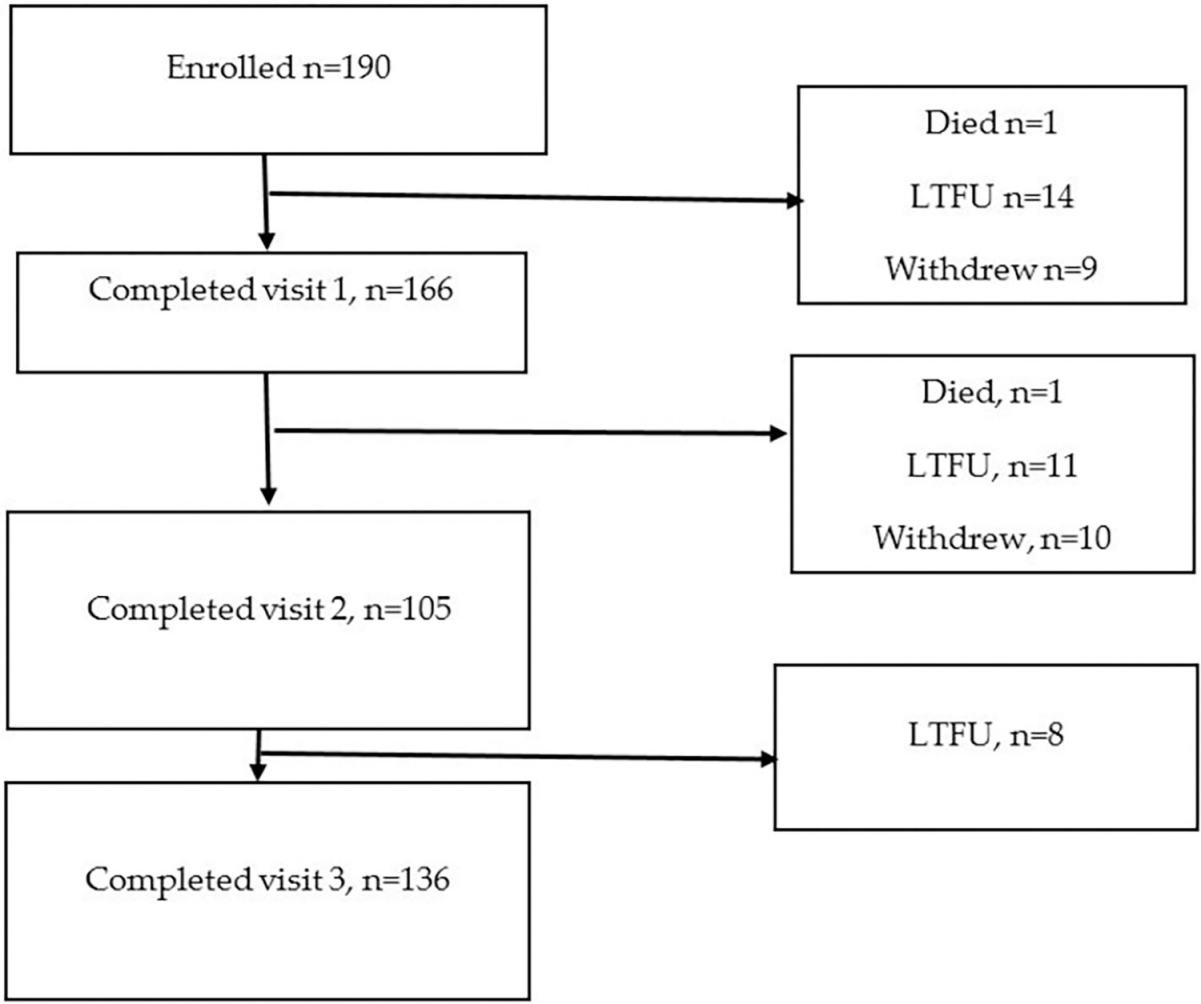
Flow of participants in the study.

Between January 01, 2022, and January 01, 2023, a total of 190 women diagnosed with preeclampsia were enrolled in the study after giving birth, of whom 59 were living with HIV on ART (Fig 1). During the follow-up period, 2 participants died, 33 were lost to follow-up, and 19 withdrew from the study. Overall, 136 (71.6% retention) participants completed the six-month follow-up: 44 living with HIV on ART and 92 without HIV. During follow-up 23 were diagnosed with chronic hypertension (8 living with HIV on ART and 15 without HIV). Similar socio-demographic characteristics (S1 Table in S1 File) were seen among those retained compared to the loss to follow-up.

### Characteristics of study participants

Table 1 shows baseline participant characteristics by the HIV serostatus at baseline. The median age was 30 years (interquartile range [IQR]: 27–34), and the majority, 158 (83.2%) were married. The largest proportion, 150 (79.0%), had secondary education, and 114 (60.0%) were in the lower wealth index quintile.

**Table 1:** Baseline sociodemographic and obstetric characteristics of participants by HIV serostatus status.

Variable	Total population n = 190(%)	HIV serostatus		P-value
		Negative, n = 131	Positive, n = 59	
Age in years, median (IQR)	30(27–34)	30(26–34)	31(27–35)	0.421
Marital status Unmarried Married	32(16.8) 158(83.2)	23(17.6) 108(82.4)	9(15.3) 50(84.8)	0.695
Education None/Primary Secondary/Tertiary	40(21.1) 150(79.0)	20(15.3) 111(84.7)	20(33.9) 39(66.1)	0.004
Wealth index Poorest/poorer/middle Richer/richest	114(60.0) 76(40.0)	74(56.5) 57(43.5)	40(67.8) 19(32.2)	0.141
Onset of preeclampsia Early (<34 weeks) Late (≥34 weeks)	93(49.0) 97(51.1)	67(51.2) 64(48.9)	26(44.1) 33(55.9)	0.367
Severity of preeclampsia Severe Mild/Moderate	15 (7.9) 175(92.1)	8(6.1) 123(93.9)	7(11.9) 52(88.1)	0.243
Pregnancy outcomes Stillbirth Preterm birth Live/term birth	25(13.2) 33(17.4) 132(69.5)	13(9.9) 28(21.4) 90(68.7)	12(20.3) 5(8.5) 42(71.2)	0.026
Parity median(IQR)	2(1–3)	2(1–3)	2(1–4)	0.070
Contraceptive use Other^a^ Intrauterine/barrier Hormonal	58(30.5) 28(14.7) 104(54.7)	32(24.4) 25(19.1) 74(56.5)	26(44.1) 3(5.1) 30(50.9)	0.005
Anxiety and depression No Yes	43(22.6) 147(77.4)	18(13.7) 113(86.3)	25(42.4) 34(57.6)	<0.001

Abbrev: IQR-interquartile range, ^a^(sterilization, lactational amenorrhea, withdrawal, fertility awareness-based methods or dual methods), p-values from Pearson Chi-square test/Fisher’s exact test/Wilcoxon Ranksum test

### Prevalence of persistent hypertension during follow-up

**Fig 2:**
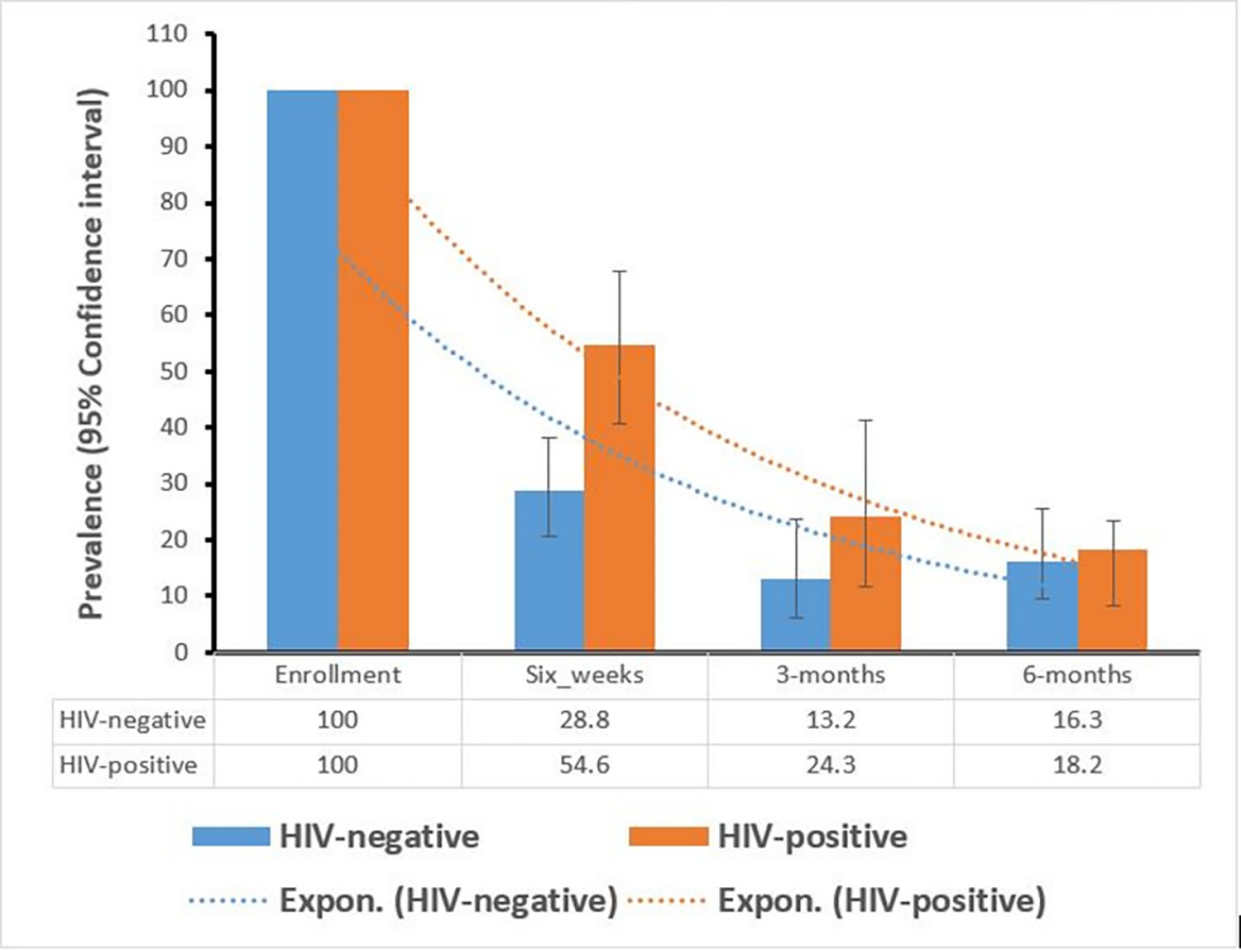
Prevalence of persistent hypertension and the trends during the follow-up visits.

The prevalence of persistent hypertension during follow-up by HIV serostatus is shown in Fig 2. Of 136 participants, 23 were hypertensive at six months after delivery (16.9%, 95% CI: 11.0–24.3). The prevalence at six weeks was higher in the HIV group than HIV-negative group (54.6% vs 28.8%, p<0.001). However, by three months (24.3% vs 13.2%, p = 0.15) and six months (18.2% vs 16.3%, p = 0.787) statistical differences were no longer observed. Analysis of trends showed a significant reduction in the proportion of participants with persistent hypertension over six months postpartum (p<0.001).

### Persistent hypertension at 6 months and participant characteristics

Table 2 shows participant characteristics by the hypertension status. The median age was 29 years (interquartile range [IQR]: 26–34), and the majority, 112 (82.4%) were married, 108(79.4%) attained at least a secondary level of education and 81(59.6%) were in the lower quintiles of wealth index. Women with and without hypertension had similar median age but differed in the median body mass index.

**Table 2:** Participants characteristics at 6 months postpartum visit by persistent hypertension.

Variable	Total population n = 136(%)	Normotensive, n = 113(%)	Hypertensive^a^, n = 23(%)	P-value
HIV serostatus Negative positive	92(67.7) 44(32.4)	77(68.1) 36(31.9)	15(65.2) 8(34.8)	0.785
Age in years (IQR)	29(26–34)	29(26–33.5)	30(27–35)	0.228
Marital status Unmarried Married	24(17.7) 112(82.4)	21(18.6) 92(81.4)	3(13.0) 20(87.0)	0.765
Education level None/Primary Secondary/Tertiary	28(20.6) 108(79.4)	24(21.2) 89(78.8)	4(17.4) 19(82.6)	0.677
Wealth index Poorest/poorer/middle Richer/richest	81(59.6) 55(40.4)	65(57.5) 48(42.4)	16(69.6) 7(30.4)	0.283
Gestational age at delivery, median(IQR)	33(30–36)	33(30–36)	34(32–36)	0.361
Onset of pre-eclampsia Early (<34 weeks) Late (≥34 weeks)	71(52.2) 65(47.8)	59(52.2) 54(47.8)	12(52.2) 11(47.8)	0.997
Severity of Pre-eclampsia Severe Mild/Moderate	9(6.6) 127(93.4)	5(4.4) 108(95.6)	4(17.4) 19(82.6)	0.044
Adverse pregnancy Stillbirth Preterm birth Live/term birth	18(13.2) 26(19.1) 92(67.7)	15(13.3) 23(20.4) 75(66.4)	3(13.0) 3(13.0) 17(73.9)	0.706
Parity median(IQR)	2(1–3)	2(1–3)	3(2–4)	0.009
Contraceptive use Other^b^ Intrauterine/barrier Hormonal	24(17.8) 17(65.7) 94(69.6)	22(19.6) 17(15.2) 73(65.2)	2(8.7) - 21(91.3)	0.035
Body mass index kg/m^2^ median(IQR)	27.3(24.2–30.4)	27.3(23.8–30.1)	27.7(25.8–30.9)	0.008
Anxiety and depression No Yes	81(59.6) 55(40.4)	72(63.7) 41(36.3)	9(39.1) 14(60.9)	0.029

Abbrev: IQR-interquartile range

^a^defined as elevated systolic blood ≥140mmHg and diastolic blood pressure≥ 90mmHg and or taking hypertension medication

^b^(sterilization, lactational amenorrhea, withdrawal, fertility awareness-based methods or dual methods)p-values from Pearson Chi-square test/Fisher’s exact test/Wilcoxon Ranksum test

### Risk factors of persistent hypertension status changes during follow-up

Table 3 shows the crude and adjusted models for risk factors of persistent hypertension status changes from each measurement time point to the other during the six-month follow-up. From the crude model, Individuals with HIV on ART had a 25% increased odds of hypertension compared to those without HIV, however, with a borderline p-value. On the other hand, the factors associated with persistent hypertension were probable depression and anxiety, body parity, severity of preeclampsia and food insecurity score.

**Table 3:** Predictors of changes in persistent hypertension^a^ over six months postpartum among women previously diagnosed with preeclampsia.

Variable	Crude odds ratio (95% CI)	p-value	Adjusted odds ratio (95% CI)	p-value
HIV serostatus Negative positive	Ref 1.25(0.99–1.58)	0.064	Ref 1.68 (1.09–2.60)	**0.019**
Age years < 30 30–39 40 or more	Ref 1.16(0.92–1.45) 1.38 (0.72–2.63)	0.195 0.332	Ref 0.91(0.59–1.41) 0.62 (0.20–1.90)	0.689 0.404
Marital status Unmarried Married	Ref 1.17(0.77–1.78)	0.258		
Education Primary/no education Secondary/Tertiary	Ref 1.04(0.71–1.53)	0.855		
Wealth index Poorest/poorer/middle Richer/richest	Ref 0.77(0.56–1.07)	0.127		
Depression and anxiety No yes	Ref 2.54(1.80–3.57)	**<0.001**	Ref 2.06 (1.35–3.14)	**0.001**
Onset of preeclampsia Early (<34 weeks) Late (>/ = 34 weeks)	Ref 1.22(0.98–1.53)	0.074		
Severity of Preeclampsia Severe Mild/Moderate	Ref 0.52(0.28–0.97)	**0.026**	0.37 (0.16–0.85)	**0.019**
Pregnancy outcomes Live/term birth Still/Preterm birth	Ref 1.01(0.72–1.43)	0.940		
Parity	1.15(1.04–1.27)	**0.002**	1.24 (1.07–1.45)	**0.005**
Food insecurity score	1.09(1.01–1.18)	**0.032**	1.11 (0.97–1.26)	**0.110**
Contraceptive use Other^b^ Intrauterine/other Hormonal	Re 1.14(0.64–2.04) 1.25(0.86–1.82)	0.421 0.403		
Body mass index kg/m^2	1.02(1.01–1.05)	**0.035**	1.03(0.99–1.06)	0.086
Time since delivery (months)	0.49 (0.43–0.54)	**<0.001**	0.51(0.46–0.57)	**<0.001**

^a^Outcome variable for the regression model (SBP>140 &/or DBP>90 coded/treatment: yes = 1, no = 0)

^b^(sterilization, lactational amenorrhea, withdrawal, fertility awareness-based methods or dual methods), adjustment variables (body mass index, age, anxiety&depression, parity, food insecurity score, severity of preeclampsia), boldface indicates statistical significance at alpha less than 0.05.

In the multivariable model, HIV on ART, probable depression and anxiety, severity of preeclampsia and parity were significantly associated with changes in hypertension status over time. Compared to HIV-negative, participants living with HIV on ART (adjusted odds ratio [AOR] = 1.68, 95% CI: 1.09–2.60) were more likely to have hypertension during the six months postpartum.

### Sensitivity analysis

From the multivariable models with continuous outcomes, HIV on ART had a significant effect on the mean descent in systolic and diastolic blood pressure (S2 Table in S1 File). On average the mean systolic and diastolic blood pressure was 4.02 and 2.72 units higher among the women living with HIV than HIV-negative women at every time point measured since delivery. There was no evidence to suggest that persistent hypertension among women living with HIV compared to HIV-negative women differ by the time since delivery (S3 Table in S1 File).

## Discussion

In a clinical sample in urban Zambia, 16.9% of postpartum women experienced persistent hypertension six months after giving birth. Those women previously diagnosed with pre-eclampsia and living with HIV on ART had 1.68-fold higher odds of persistent hypertension than their HIV-negative counterparts. Similarly, participants with increased parity and those who reported probable anxiety and depression had higher odds of persistent hypertension. On the other hand, participants with mild or moderate pre-eclampsia had lower odds of persistent hypertension than those with severe pre-eclampsia.

Persistent hypertension rate was comparable between women living with and without HIV by 6 months postpartum despite the former having higher odds of persistent hypertension at any time point during follow-up. This was corroborated by our results from sensitivity analysis where we noted a similar effect on the mean descent in systolic and diastolic blood pressure. It’s unclear about the mechanism behind the observed rates as little is known about HIV and persistent hypertension after preeclampsia in the extant literature. We speculate this could be a mismeasurement of HIV due to the complexities between HIV suppressing immunity alongside immune reconstitution through ART [39–41]. Given that HIV and ART may have complicated pathways depending on the physiological circumstance [42], further studies are warranted to fully elucidate their mechanisms in postpartum women after preeclampsia.

In the general population, some studies have reported persistent hypertension after preeclampsia; though data remains sparse [8, 9, 43]. Few studies have specifically studied HIV/ART and persistent hypertension after preeclampsia and suggested this possible association, but the limited number of participants living with HIV led to unreliable estimates [8, 44]. A recent systematic review and meta-analysis of global data demonstrated that 35% of all HIV-infected adults on ART had hypertension compared to 30% of HIV-uninfected [45]. Furthermore, results from published studies show that ART may alter various metabolic processes, leading to obesity and overweight, all of which are known risk factors for hypertension [46, 47]. Therefore, the present study’s findings align with previous findings in the general population that HIV/ART are associated with hypertension and other cardiovascular diseases [48, 49]. We speculate that HIV on ART could have an additive effect on preeclampsia, which could potentially be worsening the persistent hypertension in the postpartum period.

The prevalence of persistent hypertension at six months’ follow-up is comparable to the regional estimates. The prevalence was higher than that found in Cameroon (14.8%) [50], but lower than in Kenya (44.4%)[51] and Nigeria (62.2%) [9]. The rates in high-income countries average around 10% to 20%. For example, in the United Kingdom, the rates are 13% [52], in the USA 19% [43] and 20.3% in Switzerland, 10.8% [52] in France [53] and 17% in Japan [54]. The differences in study settings, onset, and severity of preeclampsia among studied participants could partly account for the disparity. Additionally, most high-income countries have guidelines on risk reduction for women after preeclampsia.

Participants with mild or moderate preeclampsia were less likely to have persistent hypertension than those with severe preeclampsia. These findings are consistent with similar studies from high-income countries [55, 56]. Berks and colleagues [55] demonstrated that time to resolution of hypertension postpartum in women with diagnosed preeclampsia during pregnancy increased by 1.6-fold for every 10-mm Hg increase in maximal systolic blood pressure. Additionally, a systematic review and meta-analysis showed that the risk of future hypertension later in life increased by 6.7-fold among women with severe preeclampsia [56]. The link is thought to be through the extent of endothelial dysfunction, end organ damage and elevated biomarkers of inflammation [57]. The degree of damage to the systemic organs could affect the recovery from preeclampsia.

There was a relationship between increased parity and odds of persistent hypertension. Prior studies conducted in sub-Saharan Africa and high-income countries have reported inconsistent results [44, 58]. A study by Olagbuji and colleagues in Nigeria found no evidence of an association [44]. Our study findings align with Lykke and colleagues from Denmark, who demonstrated that women with two or more pregnancies had 4.34-fold higher rates of persistent hypertension [58]. It is unclear the mechanism behind the observed association. It could be because of pregnancy’s stress on the cardiovascular system, especially in women with a history of hypertensive disorders [59]. The body undergoes significant changes during pregnancy to support the growing fetus [60]. In women with multiparity, this cumulative cardiovascular stress can contribute to a higher risk of persistent hypertension after giving birth and in years to come [61].

There is a lack of studies exploring the link between anxiety, depression and persistent hypertension in women previously diagnosed with pre-eclampsia. A systematic review of studies from LMICs reported an association between food insecurity and depression in the general population [62] and similar findings have been reported among pregnant women in Ethiopia and Uganda [63–65]. It is plausible that the same underlying drivers that lead to persistent hypertension may simultaneously underpin anxiety and depression, including economic instability, housing insecurity, or exposure to violence [66]. Our findings add to this growing literature by identifying the link between anxiety, depression and persistent hypertension in postpartum women after preeclampsia.

Postpartum care for women living with HIV should consider long-term cardiovascular risk assessment before discharge from the hospital. This is important, particularly in LMICs that share a disproportionate burden of hypertension and HIV. The risk for each individual should determine the length of postpartum follow-up based on the assessment, which should include family history and cardiovascular risk scores.

### Strengths and limitations

This is among the first studies to explore persistent hypertension after preeclamptic pregnancies in a high HIV prevalence setting. If confirmed by other studies, postpartum monitoring may serve as an early entry point for long-term hypertensive care among high-risk individuals. However, these findings should be interpreted in the context of some limitations. We could not assess the independent effect of ART since all women living with HIV were on ART. Furthermore, even though most similar studies have a shorter follow-up, six months may be too short for cardiovascular events to be observed. Therefore, there is a need for studies with a longer follow-up period to examine how hypertension resolves in this population and the independent effect of ART.

## Conclusion

We found an elevated risk of persistent hypertension among participants living with HIV on ART. Peripartum patients in HIV-endemic settings may benefit from timely detection and treatment interventions to improve health outcomes. Timely detection and treatment of persistent hypertension could have lasting impacts on maternal health.

## Supporting information

S1 Checklist Strobe checklist. (PDF)

S2 Checklist Inclusivity in global research. (PDF)

S3 Checklist PLOSOne_human_subjects_research_checklist _MM. (PDF)

S1 Data set Data set used for the study findings. (CSV)

S1 File (DOCX)

S2 File Questionnaires. (PDF)
