# Supplementary material for: Persistent hypertension among postpartum women with comorbid HIV and preeclampsia in Zambia
Source: PLoS One. 2024 Sep 4;19(9):e0309915. doi: 10.1371/journal.pone.0309915 (PMC11373822; doi:10.1371/journal.pone.0309915)
Supplement: S1 File — (DOCX) [file pone.0309915.s005.docx]

Table S1. Cross-tabulations: Baseline characteristics and loss to follow-up

| **Variable** | **Not LTFU** | **LTFU** | **p-value** |
| --- | --- | --- | --- |
| Age in years, median (IQR) | 31(28-33) | 29(26-34) | 0.227 |
| Marital status  Unmarried  Married | 9(16.7)  45(83.3) | 23(16.9)  113(83.1) | 0.968 |
| Education  None/Primary  Secondary/Tertiary | 12(22.2)  42(77.8) | 28(20.6)  108(79.4) | 0.803 |
| Wealth index  Poorest/poorer/middle  Richer/richest | 28(58.3)  20(41.7) | 74(60.7)  48(39.3) | 0.781 |
| Onset of preeclampsia  Early (<34 weeks)  Late (≥34 weeks) | 24(44.4)  30(55.6) | 69(50.7)  67(49.3) | 0.434 |
| Severity of preeclampsia  Severe  Mild/Moderate | 5(9.3)  49(90.7) | 8(5.9)  127(94.1) | 0.413 |
| Adverse pregnancy outcomes  Other  Preterm birth/stillbirth | 40(74.1)  14(25.9) | 92(68.7)  42(31.3) | 0.462 |
| Parity median(IQR) | 1(1-3) | 2(1-3) | 0.081 |

LTFU- Loss to follow up, IQR- interquartile range, p-values from Pearson Chi-square test

Table S2. Multivariable models for the predictors of blood pressure levels

| **Variable** | **Sytolic blood pressure, mmHg** | | **Diastolic blood pressure, mm/Hg** | |
| --- | --- | --- | --- | --- |
|  | **Adjusted β Coefficient (95% CI)** | **p-value** | **Adjusted β Coefficient (95% CI)** | **p-value** |
| HIV serostatus  Negative  positive | Ref  4.04 (0.90, 7.18) | **0.012** | Ref  2.79 (0.48, 5.10) | **0.018** |
| Age years  < 30  30-39  40 or more | Ref  0.15(-3.04, 3.34)  0.63(-7.78, 9.04) | 0.926  0.883 | -0.46(-2.79, 1.88)  -7.53(-13.81, -1.23) | 0.701  **0.019** |
| Depression and anxiety  No  yes | Ref  3.42(0.39, 6.46) | **0.027** | 1.85(-0.23, 3.94) | 0.083 |
| Severity of Preeclampsia  Severe  Mild/Moderate | Ref  -1.54(-7.34, 4.26) | 0.602 | Ref  -6.84(-11.07, -2.62) | **0.001** |
| Parity | 0.70(-0.37, 1.78) | 0.201 | 0.79(0.01, 1.57) | **0.047** |
| Food insecurity score | -0.05(-0.91, 0.80) | 0.900 | 0.45(-0.14, 1.06) | 0.134 |
| Body mass index kg/m^2 | 0.35(0.07, 0.64) | **0.015** | 0.35(0.15, 0.56) | **0.001** |
| Time since delivery (months) | -5.14(-5.71, -4.55) | **<0.001** | -1.56(-2.03, -1.10) | **<0.001** |

Abbrev: β-regression coefficient, 95% CI- 95% confidence intervals, the models were fitted with systolic and diastolic blood pressure as continuous outcomes. We used the GEE model with unstructured correlation and identify link function. Boldface indicates statistical significance at p<0.05

Table S3 Interaction between HIV/ART and time since delivery

| **Variable** | **Crude odds ratio (95% CI)** | **p-value** | **Adjusted odds ratio^a^ (95% CI)** | **p-value** |
| --- | --- | --- | --- | --- |
| HIV serostatus  Negative  positive | Ref  1.71 (0.94- 3.09) | 0.078 | Ref  2.02 (1.08-3.81) | **0.029** |
| Time since delivery (months) | (0.48 0.42-0.55) |  | 0.53 (0.46-0.61) | **<0.001** |
| HIV#time since delivery  Negative  positive | Ref  1.01(0.79-1.27) | 0.985 | Ref  0.92(0.72-1.16) | 0.466 |

In the crude model we included on HIV and time since delivery. In the adjusted model: adjustment variables (body mass index, age, anxiety&depression, parity, food insecurity score, severity of preeclampsia), Boldface indicates statistical significance at p<0.05
