# Supplementary material for: Persistent hypertension among postpartum women with comorbid HIV and preeclampsia in Zambia
Source: PLoS One. 2024 Sep 4;19(9):e0309915. doi: 10.1371/journal.pone.0309915 (PMC11373822; doi:10.1371/journal.pone.0309915)
Supplement: S2 File — (PDF) [file pone.0309915.s006.pdf]

Questionnaires

Research title: Persistent hypertension among postpartum women with comorbid HIV and preeclampsia in Zambia

Visit number _____________Participant id ________________ Date___________________

Note! Before answering the questionnaires on return visits, participants should reread the information sheet as a guide to this questionnaire.

Instructions:
Please answer the following questions for each part of the questionnaire. Your responses are strictly confidential and anonymous. It will take you 20 to 30 minutes to complete. The questionnaire has 5 parts (Part A to E). Each part addresses a specific characteristic to help us classify you to a correct group in our study. You may skip questions or parts you do not feel comfortable with. You may discontinue participation/ answering the questionnaire at any time without penalty, or loss of benefits to which you are otherwise entitled. Furthermore, there is no requirement to provide a reason for withdrawing and any data collected from you will in default be destroyed unless you expressly consent to its retention.

Please fill in the following relating to you so that we can know more about you. If this is a return visit and nothing has changed, kindly indicate so in the question column and proceed to part A

Appendix 3. Questionnaires

Visit number ____________Participant id _______________ Date__________________

Note! Before answering the questionnaires on return visits, participants should reread the information sheet as a guide to this questionnaire.

Instructions:
Please answer the following questions for each part of the questionnaire. Your responses are strictly confidential and anonymous. It will take you 20 to 30 minutes to complete. The questionnaire has 5 parts (Part A to E). Each part addresses a specific characteristic to help us classify you to a correct group in our study. You may skip questions or parts you do not feel comfortable with. You may discontinue participation/ answering the questionnaire at any time without penalty, or loss of benefits to which you are otherwise entitled. Furthermore, there is no requirement to provide a reason for withdrawing and any data collected from you will in default be destroyed unless you expressly consent to its retention.

Please fill in the following relating to you so that we can know more about you. If this is a return visit and nothing has changed, kindly indicate so in the question column and proceed to part A

No.	Question	Response options (tick/write what applies to you)
1	Residential address	____
2	Email address	____
3	Cell number	_____________________________________
4	Marital status	☐married ☐ single ☐widowed ☐never married ☐other
5	Education	☐no education ☐primary ☐secondary ☐tertiary
6	Housing type	☐ unplanned ☐ low cost ☐medium cost ☐high cost
7	Household income (Kwacha)	____
8	Religion	☐Christian ☐Muslim ☐hindu ☐other
9	Household size (# who stay in the house)	____________________

Part A: An alcohol screening tool - Self-Report Version

Because alcohol use can affect your health and can interfere with certain medications and treatments, it is important that we ask some questions about your use of alcohol. Your answers will remain confidential so please be honest. Place an X in one box that best describes your answer to each question. You can skip this part if you feel uncomfortable answering any questions

Do you drink any form of alcohol? ☐ yes ☐no
If no, then move to part B

Circle the response option that best suits you

AUDIT-C Question	Response options					
	**0**	**1**	**2**	**3**	**4**	
How often do you have a drink containing alcohol?	Never or	Monthly	less 2-4 times per month	2-3 times per week	4+ times per	
How many drinks containing alcohol do you drink on a typical day when you are drinking?	1-2	3-4	5-6	7-9	10+	
How often have you had 6 or more drinks on one occasion in the last year?	Never	Less than monthly	Monthly	Weekly	Daily or almost daily	
**(Score) To be completed by research staff**						

Part B: Smoking: Nicotine Dependence for Daily and Nondaily Smokers – Short Form 4a

1. Do you smoke any form of cigarettes? □ yes □no

If no, then move to part C

2. How many cigarettes do you smoke?

A) > one cigarette/day and lasts more than six months

B) I stopped smoking

3. Please respond to each question or statement by marking one box per row

Question	1.Never	2.Rarely	3.Sometimes	4.Often	5.Always
When I haven't been able to smoke for a few hours, the craving gets intolerable					
find myself reaching for cigarettes without thinking about it					
I drop everything to go out and buy cigarettes					
I smoke more before going into a situation where smoking is not allowed					
**(Score) To be completed by research staff**					

Part C: Hospital Anxiety and Depression Scale (HADS)

Tick the box beside the reply that is closest to how you have been feeling in the past week. Don't take too long over your replies: your immediate is best.

D	A		D	A	
		**I feel tense or 'wound up':**			**I feel as if I am slowed down:**
	3	Most of the time	3		Nearly all the time
	2	A lot of the time	2		Very often
	1	From time to time, occasionally	1		Sometimes
	0	Not at all	0		Not at all
		**I still enjoy the things I used to enjoy:**			**I get a sort of frightened feeling like 'butterflies' in the stomach:**
0		Definitely as much		0	Not at all
1		Not quite so much		1	Occasionally
2		Only a little		2	Quite often
3		Hardly at all		3	Very often
		**I get a sort of frightened feeling as if something awful is about to happen:**			**I have lost interest in my appearance**
	3	Very definitely and quite badly	3		Definitely
	2	Yes, but not too badly	2		I don't take as much care as I should
	1	A little, but it doesn't worry me	1		I may not take quite as much care
	0	Not at all	0		I take just as much care as ever
		**I can laugh and see the funny side of things:**			**I feel restless as I have to be on the move**
0		As much as I always could		3	Very much indeed
1		Not quite so much now		2	Quite a lot
2		Definitely not so much now		1	Not very much
3		No at all		0	Not at all
		**Worrying thoughts go through my mind:**			**I look forward with enjoyment to things:**
	3	A great deal of the time	0		As much as I ever did
	2	A lot of the time	1		Rather less than I used to
	1	From time to time, but not too often	2		Definitely less than I used to
	0	Only occasionally	3		Hardly at all
		**I feel cheerful**			**I get sudden feelings of panic**
3		Not at all		3	Very often indeed
2		Not often		2	Quite often
1		Sometimes		1	Not very often
0		Most of the time		0	Not at all
		**I can sit at ease and feel relaxed:**			**I can enjoy a good book or radio or TV program:**
	0	Definitely	0		Often
	1	Usually	1		Sometimes
	2	Not often	2		Not often
	3	Not at all	3		Very seldom

Please check you have answered all the questions

For research staff use:

Total score: Depression (D)_______________ Anxiety (A)________________

Part D: Food security (Household Hunger Scale)

This section will ask you questions on how secure you are with food requirements for yourself and family. You may respond by ticking/ circling your best response option provided.

No.	Question	Response Option (circle your choice)	Code
Q1	In the past [4 weeks/30 days], was there ever no food to eat of any kind in your house because of lack of resources to get food?	0 = No (Skip to Q2)<br>1 = Yes	|___|
Q1a	How often did this happen in the past [4 weeks/30 days]?	1 = Rarely (1–2 times)<br>2 = Sometimes (3–10 times)<br>3 = Often (more than 10 times)	|___|
Q2	In the past [4 weeks/30 days], did you or any household member go to sleep at night hungry because there was not enough food?	0 = No (Skip to Q3)<br>1 = Yes	|___|
Q2a	How often did this happen in the past [4 weeks/30 days]?	1 = Rarely (1–2 times)<br>2 = Sometimes (3–10 times)<br>3 = Often (more than10 times)	|___|
Q3	In the past [4 weeks/30 days], did you or any household member go a whole day and night without eating anything at all because there was not enough food?	0 = No (Skip to the next section)<br>1 = Yes	|___|
Q3a	How often did this happen in the past [4 weeks/30 days]?	1 = Rarely (1–2 times)<br>2 = Sometimes (3–10 times)<br>3 = Often (more than 10 time	|___|
		**(Score) To be completed by research staff**	

Part E: Socioeconomic status (Wealth index assessment) using principal component analysis (PCA)

This part will assess your wealth index. Please select all that applies to you by ticking the option in the response column

Question No.	Characteristic	Response options
1	Source of drinking water	|___|Piped into dwelling, |___| piped into compound, |___|piped to neighbour, |___| piped to kiosk, |___|public tap, |___|borehole, |___|protected dug well, |___|unprotected dug well, |___|protected spring water, |___|unprotected spring water, |___|rainwater collection, |___| tanker-truck, |___|cart with small tank/drum, |___|surface water, |___|bottled water |___|, other
2	Type of toilet	|___|Flush to sewer, |___|flush to septic tank, |___|flush to pit latrine, |___|flush to somewhere else, |___|flush to unknown, |___|ventilated improved pit latrine, |___|pit latrine with slab, |___| open pit latrine, |___|composting toilet, |___|bucket, hanging latrine, |___| no facility
3	Type of floor	|___|earth/sand, |___|dung, |___|wood planks, |___|parquet or polished wood, |___|vinyl or asphalt strips, |___|ceramic tiles, |___|cement, |___|carpet
4	Type of roof	|___|No roof, |___|grass/thatch, |___|dung/mud, |___|iron sheet, |___|tin cans, |___|asbestos sheet, |___|concrete, |___|tiles
5	Type of wall	|___|No walls, |___|cane/palm/trunks, |___|dirt, |___|bamboo with mud, |___|stone with mud, |___|uncovered adobe, |___|plywood, |___|cardboard, |___|reused wood, |___|cement, |___| stone with lime/cement, |___|bricks, |___|cement blocks, |___| covered adobe, |___| wood planks/shingles, |___|others
6	Type of cooking fuel	|___|Electricity, |___|liquefied petroleum gas (LPGAs), |___|Natural gas, |___|biogas, |___| kerosene, |___| coal, |___| charcoal, |___| wood, |___| straw/shrubs/grass
7	Use of electricity	|___| Yes, |___| No
8	Possession of radio	|___| Yes, |___| No
9	Possession of TV	|___| Yes, |___| No
10	Possession of refrigerator	|___| Yes, |___| No
11	Possession of Washing machine	|___| Yes, |___| No
12	Availability of landline phone	|___| Yes, |___| No
13	Possession of mobile phone	|___| Yes, |___| No
14	Possession of bicycle	|___| Yes, |___| No
15	Possession of motorcycle/ scooter	|___| Yes, |___| No
16	Possession of watch	|___| Yes, |___| No
17	Possession of computer	|___| Yes, |___| No
18	Possession of car/ truck	|___| Yes, |___| No
19	Possession of animal drawn cart	|___| Yes, |___| No
20	Type household dwelling	|___| Own, |___| Rent, |___| Squatter/stay for free/others
21	Help from employed person	|___| Yes, |___| No
22	Agricultural land ownership	|___| Yes, |___| No
23	Livestock ownership	|___| Yes, |___| No

This is the end of this questionnaire. Thank you for your responses.

Date questionnaire completed________________________________
